# Vortex solitons in topological disclination lattices

**DOI:** 10.1515/nanoph-2023-0790

**Published:** 2024-01-22

**Authors:** Changming Huang, Ce Shang, Yaroslav V. Kartashov, Fangwei Ye

**Affiliations:** Department of Physics, Changzhi University, Changzhi, Shanxi 046011, China; King Abdullah University of Science and Technology (KAUST), Physical Science and Engineering Division (PSE), Thuwal 23955-6900, Saudi Arabia; Institute of Spectroscopy, Russian Academy of Sciences, 108840, Troitsk, Moscow, Russia; School of Physics and Astronomy, Shanghai Jiao Tong University, Shanghai 200240, China

**Keywords:** vortex solitons, topological disclination lattices, stability, propagation dynamics

## Abstract

The existence of thresholdless vortex solitons trapped at the core of disclination lattices that realize higher-order topological insulators is reported. The study demonstrates the interplay between nonlinearity and higher-order topology in these systems, as the vortex state in the disclination lattice bifurcates from its linear topological counterpart, while the position of its propagation constant within the bandgap and localization can be controlled by its power. It is shown that vortex solitons are characterized by strong field confinement at the disclination core due to their topological nature, leading to enhanced stability. Simultaneously, the global discrete rotational symmetry of the disclination lattice imposes restrictions on the maximal possible topological charge of such vortex solitons. The results illustrate the strong stabilizing action that topologically nontrivial structures may exert on excited soliton states, opening new prospects for soliton-related applications.

## 1 Introduction

Vortex solitons are localized, self-trapped states with nonzero orbital angular momentum. They were encountered in various physical systems such as nonlinear optical materials, Bose–Einstein condensates, polariton condensates, and plasmas [1]–[9]. Since phase singularity in vortex is a topologically stable object persisting even in the presence of perturbations, such states have important applications in tweezers [10], vortex microlasers [11], and information encoding [12], [13]. Vortex solitons are ideal for optical logic gates in all-optical computing and communication [14]. At the same time, being higher-order excited nonlinear states, they are prone to various dynamical instabilities. Different approaches have been proposed to stabilize them (see reviews [2], [3]) that include the utilization of competing or nonlocal nonlinearities, rapid parameter variations, spin–orbit coupling, and various optical potentials including periodic lattices [15]–[19]. Remarkably, when such potentials possess discrete rotational symmetry, they impose restrictions on the available charges of supported vortex solitons [20], [21], [22]. While it is predicted that semi-vortex solitons can emerge in the bulk of topological lattices in the continuum limit [23], [24] and nonvortical solitons sustained by continuous Jackiw–Rossi-like distortion [25] were reported, strongly localized vortex solitons in potentials belonging to the class of topological insulators have not been studied to our knowledge.

The remarkable property of topological insulators is the existence of localized states at their edges or corners, which are protected by the system’s topology. These states have energies that fall within the forbidden topological gaps. The theory of quantized polarization [26], [27] connecting the topological properties of bulk bands in such structures with the appearance of edge states has recently been extended from dipole to multipole moments, showcasing the development from first-order topological insulators [28], [29] to higher-order ones [30]–[37]. The bulk-boundary correspondence in these systems may be characterized by a codimension ranging from one to higher. Furthermore, topological systems exhibit a rich variety of nonlinear phenomena that acquire unique features due to their topological nature. These phenomena include the formation of topological solitons [38]–[51], lasing in topological states [52], [53], [54], [55], enhanced generation of higher harmonics [56], nonlinear Thouless pumps [57]–[61], among others. Despite the exciting opportunities provided by topological systems for the formation of fundamental topological solitons, the absence of discrete rotational symmetry at the boundaries of most of such topological structures poses a significant challenge for creating topological vortex solitons.

This work aims to introduce optical vortex states in a nonlinear version of recently discovered topological disclination lattices [62]–[68] (also explored in acoustic realizations [69], [70], [71]). We find that these solitons form at the disclination core of the lattices with different discrete rotational symmetries, that they are thresholdless because they bifurcate from linear topological disclination modes, and that lattice topology may grant enhanced stability to such states. Our results not only provide the first example of compact nonlinear vortex state in a topological system but also show that such states in disclination lattices can possess topological charges forbidden in systems based on periodic lattices, such as square or honeycomb ones.

## 2 Disclination lattices

**Figure 1:**
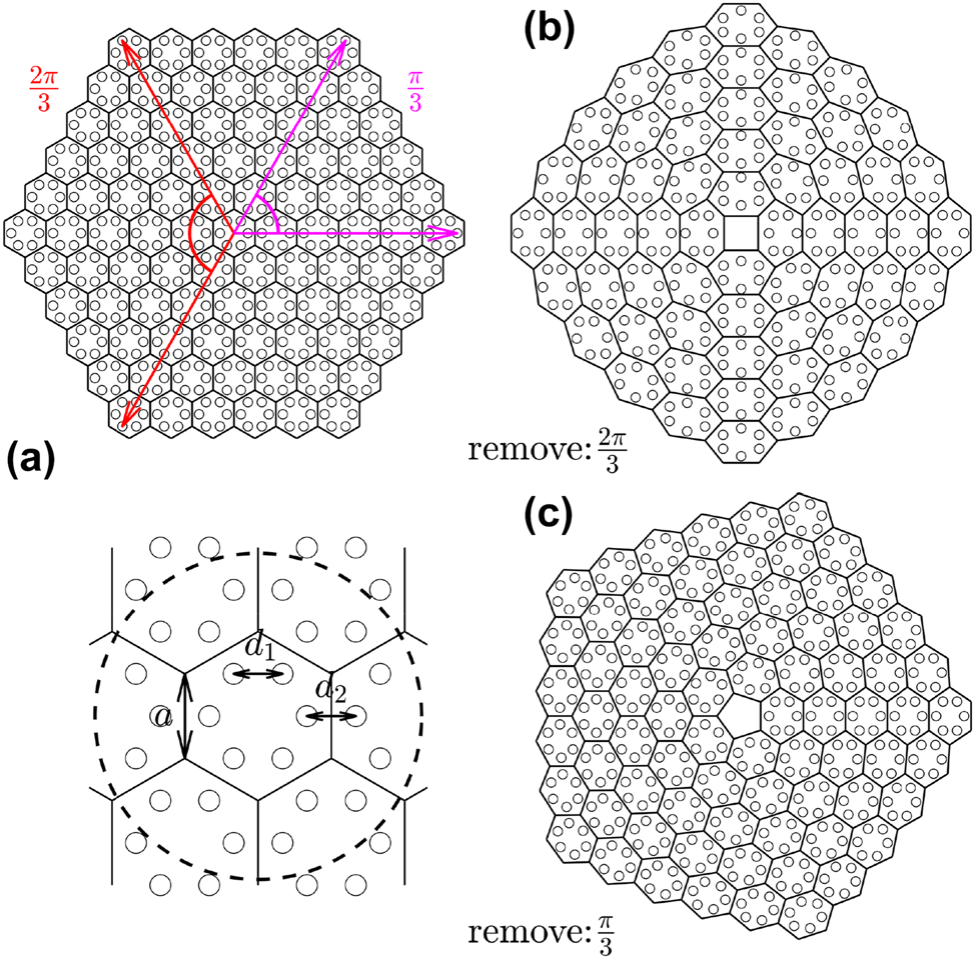
Illustration of the method of construction of disclination lattice. (a) Original hexagonal lattice. (b) and (c) Disclination lattices with $C_{4}$ (or $C_{5}$ ) symmetry obtained by removing a 2*π*/3 (or *π*/3) sector from the original hexagonal lattice.

To generate a disclination lattice, we employ the Volterra process, which involves removing or inserting a *nπ*/3 sector from a hexagonal sample [62]. This process generates a disclination with a Frank angle of ±*nπ*/3, resulting in structures with $C_{6\pm n}$ discrete rotational symmetry, as shown in Figure 1. For the optical realization, we suppose that original $C_{6}$ sample is composed of Gaussian waveguides with the depth *p* and width *w* [see the inset below Figure 1(a)], *a* is the length of the lattice cell, *d*_1_ is the intracell waveguide spacing, *d*_2_ is the intercell waveguide spacing. We set *a* ≡ 3, *p* = 8, and *w* = 0.5. Varying parameter *γ* = *d*_1_/*d*_2_ quantifies relative strength of intra- and intercell coupling. As shown in Figure 1(b), one can obtain a lattice with $C_{4}$ discrete rotational symmetry by removing the 2*π*/3 sector from the hexagonal structure and gluing the remaining parts together. Similarly, by inserting the 2*π*/3 sector into a hexagonal structure, one can obtain a lattice with $C_{8}$ symmetry (see Supplementary Materials [72]). Disclination lattices with $C_{5}$ [Figure 1(c)] and $C_{7}$ rotational symmetry are obtained by removing or inserting the *π*/3 sector. In all cases, one can see the formation of a disclination core in the center of the lattice. Remarkably, this procedure allows to construct structures with discrete rotational symmetry not attainable in usual periodic lattices.

The propagation of light beam along the *z*-axis in the disclination lattice created in the cubic nonlinear medium can be described by the nonlinear Schrödinger equation for dimensionless field amplitude Ψ:

(1) $$i\frac{\partial \Psi }{\partial z}=-\frac{1}{2}\nabla^{2}\Psi -V\left(x,y\right)\Psi -g|\Psi {|}^{2}\Psi ,$$

where ∇ = (*∂*/*∂x*, *∂*/*∂y*), the transverse *x*, *y* and longitudinal *z* coordinates are normalized to the characteristic scale *r*_0_ = 10 μm and diffraction length $kr_{0}^{2}\approx 1.14\;mm$ , respectively, *k* = 2*πn*/*λ* is the wavenumber at *λ* = 800 nm, *n* ≈ 1.45 is the background refractive index, the dimensionless intensity |Ψ|^2^ corresponds to $I=n|\Psi {|}^{2}/k^{2}r_{0}^{2}|n_{2}|$ (in fused silica *n*_2_ ≈ 2.7 × 10^−20^ m^2^/W), *g* = +1 (*g* = −1) corresponds to focusing (defocusing) nonlinearity. The function $V\left(x,y\right)=p\sum_{m,n}e^{-\left[{\left(x-x_{m}\right)}^{2}+{\left(y-y_{n}\right)}^{2}\right]/w^{2}}$ describes a disclination lattice, where lattice depth $p=k^{2}r_{0}^{2}\delta n/n$ is proportional to the refractive index contrast *δn* (*p* = 8 corresponds to *δn* ∼ 9 × 10^−4^), (*x*_*m*_, *y*_*n*_) are the coordinates of the waveguides in disclination structure, and *w* = 0.5 (corresponding to 5 μm) is the waveguide width. Such structures can be inscribed in nonlinear transparent dielectrics using the fs-laser writing technique [43], [44]. Straight waveguides in such lattices may exhibit low propagation losses not exceeding 0.1 dB/cm at *λ* = 800 nm, enabling observation of solitons and rich nonlinear dynamics on typical sample lengths of 20 cm.

## 3 Linear disclination modes

**Figure 2:**
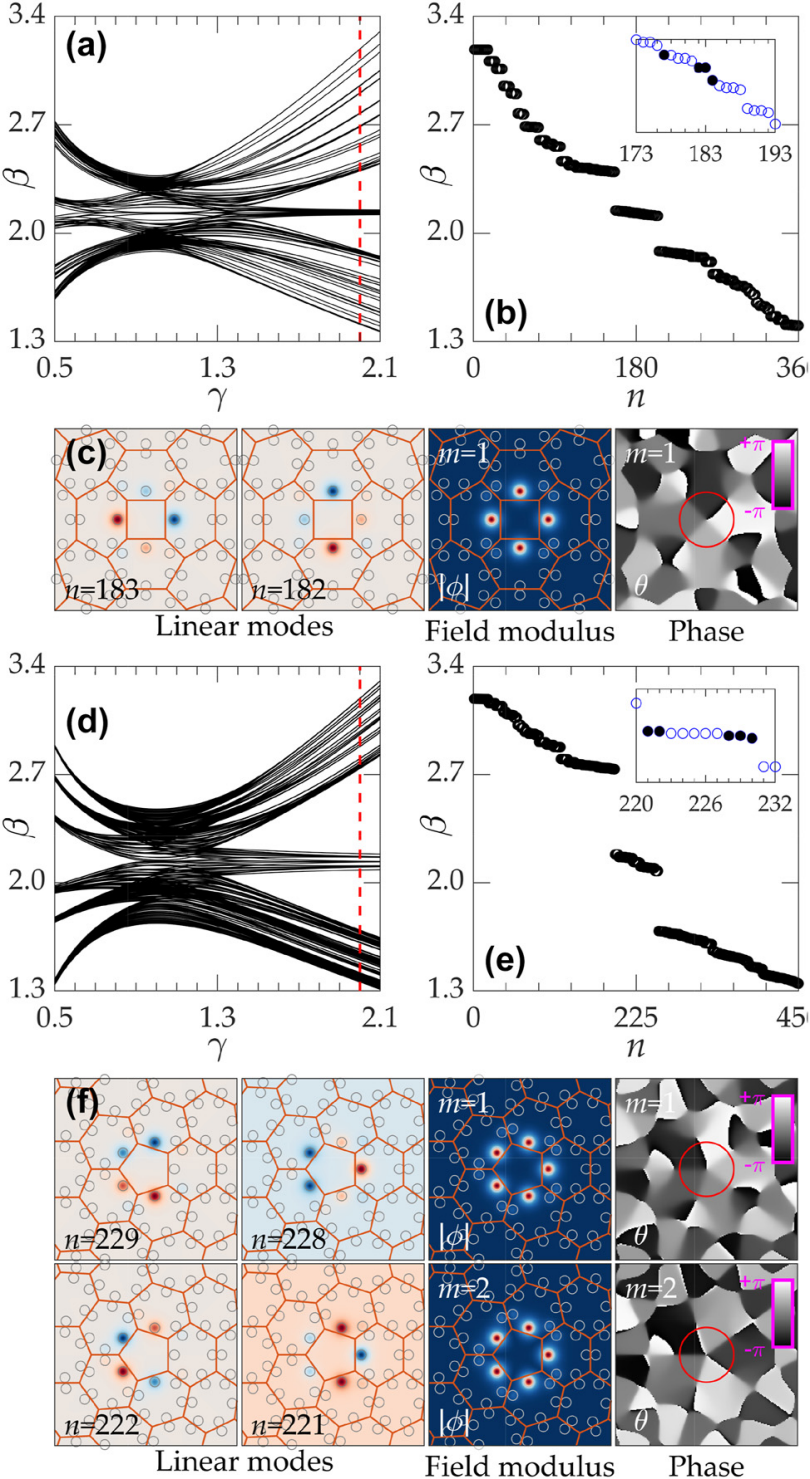
Disclination and vortex modes in $C_{4}$ and $C_{5}$ Lattices. (a) Dependencies *β*(*γ*) for $C_{4}$ lattice. (b) Eigenvalues at *γ* = 2 corresponding to the red dashed line in (a). The inset in (b) shows the indices of four linear disclination modes localized at the disclination core. (c) Two localized degenerate modes supported by the $C_{4}$ lattice and field modulus and phase distributions of vortex mode with *m* = 1 that they generate. (d) Dependencies *β*(*γ*) for $C_{5}$ lattice. (e) Eigenvalues at *γ* = 2 corresponding to the red dashed line in (d). Localized disclination modes are marked with black solid dots in the inset of (e). (f) Linear vortex mode with *m* = 1 composed from the degenerate states *ϕ*_*n*=228,229_ and that with *m* = 2 composed from the degenerate states *ϕ*_*n*=221,222_. Red lines in (c) and (f) depict lattice cells, while circles show waveguides.

To understand the structure of possible vortex solitons in disclination lattices, we first examine their linear spectra, which can be obtained by setting *g* = 0 in Eq. (1) and searching the eigenmodes of the form Ψ(*x*, *y*, *z*) = *ϕ*(*x*, *y*)e^i*βz*^, where *β* is the propagation constant (eigenvalue) and the real function *ϕ*(*x*, *y*) describes the modal field. The dependencies of the eigenvalues *β* on the distortion parameter *γ* is shown for disclination lattices with $C_{4}$ and $C_{5}$ discrete rotational symmetry in Figure 2(a) and (d), respectively. In the nontrivial topological regime *γ* > 1 (see [72] and [62]–[67] for topological characterization of disclination lattices), localized modes emerge on the central disclination core. The spectral gap, where disclination modes appear, opens for sufficiently large *γ* and increases with *γ* leading to stronger localization of disclination states. For a fixed value of *γ* = 2 [indicated by the red dashed lines in Figure 2(a) and (d)], the gap of the $C_{4}$ lattice corresponds to *β* ∈ [1.884, 2.397], while in $C_{5}$ lattice, it corresponds to *β* ∈ [1.687, 2.734]. There are no localized modes in the nontopological regime *γ* < 1.

The disclination lattice with $C_{N}$ symmetry supports *N* topological disclination modes, some of which can be degenerate [see insets of Figure 2(b) and (e)]. Linear combination of degenerate modes can produce vortex disclination states. In the lattice with $C_{4}$ symmetry, only one set of degenerate states *ϕ*_*n*=182,183_ with identical eigenvalues was found (here *n* is the index of disclination state depending on the structure size), whose linear combination *ϕ*_*n*=182_ ± *iϕ*_*n*=183_ produces single-charge (*m* = ±1) vortex mode, whose field modulus and phase distributions are shown in Figure 2(c) (notice that this mode occupies all four sites of the disclination core). The topological charge of the mode is defined using the formula: $m=\frac{1}{2\pi }\text{Im}\int_{0}^{2\pi }\partial \phi \left(r_{0},\varphi \right)/\partial \varphi /\phi \left(r_{0},\varphi \right)d\varphi$ , where *r*_0_ is a fixed small radius, and *φ* is the azimuthal angle.

In the spectrum of disclination lattice with $C_{5}$ symmetry [see the example in Figure 2(e) for *γ* = 2], one can identify two pairs of degenerate states *ϕ*_*n*=228,229_ and *ϕ*_*n*=221,222_. The combination *ϕ*_*n*=229_ ± *iϕ*_*n*=228_ yields *m* = ±1 disclination vortex, while combination *ϕ*_*n*=222_ ± *iϕ*_*n*=221_ yields *m* = ±2 state, both of them are strongly localized on the disclination core for this value of *γ*, see profiles in Figure 2(f). $C_{7}$ and $C_{8}$ structures with higher rotational symmetries (see [72]) support disclination vortices with charges up to *m* = ±3, and so on, so that available charge of disclination vortex in $C_{N}$ lattice is given by *m* < *N*/2 (for even *N*) and *m* < (*N* + 1)/2 (for odd *N*). It should be stressed that topological vortex modes on disclination are rather robust objects that persist even in the presence of disorder in the lattice. To illustrate this, we show that such vortices survive upon propagation in the lattice, where depths of individual waveguides were allowed to change randomly within the interval [*p* − *δ*, *p* + *δ*], with *δ* ≪ *p* (see [72]).

## 4 Vortex solitons in disclination lattices

We now consider vortex solitons governed by Eq. (1) with *g* ≠ 0. Because linear spectrum is characterized by the gap with topologically protected linear disclination modes in it, in-gap vortex solitons can bifurcate from such states in both focusing and defocusing media, and, importantly, nonlinearity can be used to control the location of such nonlinear states in the gap. To study properties of such “excited” vortex solitons, clearly different from their fundamental counterparts [73], we search for solutions of Eq. (1) in the form Ψ(*x*, *y*, *z*) = *ϕ*(*x*, *y*)e^i*βz*^, where *ϕ*(*x*, *y*) = *ϕ*_*r*_(*x*, *y*) + *iϕ*_*i*_(*x*, *y*) is the complex function describing vortex soliton profile, while *β* is the nonlinear propagation constant. The real *ϕ*_*r*_ and imaginary *ϕ*_*i*_ parts satisfy coupled nonlinear equations

(2) $$-\frac{1}{2}\nabla^{2}\phi_{r,i}-V\phi_{r,i}-g\left(\phi_{r}^{2}+\phi_{i}^{2}\right)\phi_{r,i}+\beta \phi_{r,i}=0,$$

that can be solved using a standard Newton iteration method with a targeted error tolerance 10^−8^.

**Figure 3:**
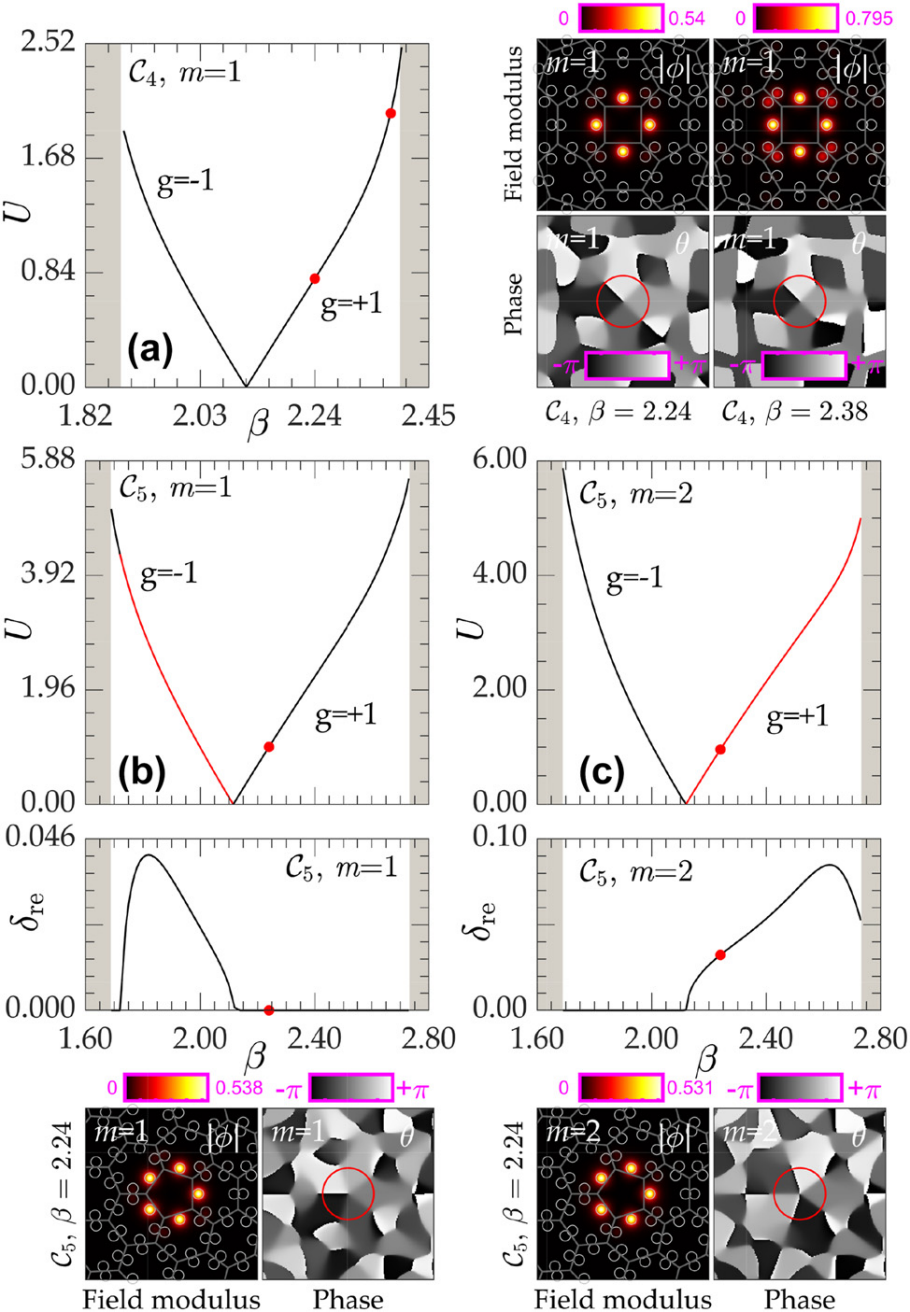
Power *U* and maximal (among all perturbations) real part of perturbation growth rate *δ*_re_ versus propagation constant *β* for vortex soliton families in disclination lattices with focusing and defocusing nonlinearity. (a) *m* = 1, $C_{4}$ lattice, (b) *m* = 1, $C_{5}$ lattice, and (c) *m* = 2, $C_{5}$ lattice. Field modulus and phase distributions of typical vortex solitons corresponding to the red dots are shown at the right (a) or at the bottom (b and c) of panels with *U*(*β*) curves. The gray regions in the *U*(*β*) plot illustrate bulk bands. Stable and unstable branches are indicated by black and red lines, respectively.

The families of vortex solitons in disclination lattices with $C_{4}$ and $C_{5}$ symmetry are presented in Figure 3 in the form of dependencies of soliton power $U=\int_{-\infty }^{+\infty }\int_{-\infty }^{+\infty }|\phi \left(x,y\right){|}^{2}dxdy$ on the propagation constant *β*. The formation mechanism of vortex solitons in the topological gap is similar to that of gap solitons, with the only difference being that gap solitons bifurcate from the edge of the allowed band, while our disclination vortices bifurcate from the topological state within the depth of the gap. For a given lattice $C_{N}$ , we present the results for focusing (*g* = +1) and defocusing (*g* = −1) medium in the same plot. It can be observed that the sign of nonlinearity determines the direction of bifurcation: in focusing medium, soliton power *U* increases with the increase in *β*, while in defocusing medium, *U* increases with a decrease in *β*. Power vanishes at the bifurcation point, which corresponds to the *β* value associated with degenerate linear modes producing vortex of a given charge *m* (thus, such solitons are thresholdless), and it notably grows as *β* approaches upper or lower edges of the gap. While well within the gap vortex solitons are localized mostly on *N* sites of disclination core, close to the gap edge they start expanding into the lattice bulk and, if *β* shifts into the band, coupling with bulk modes occurs, and the soliton delocalizes. This is illustrated in Figure 3(a) where profiles of two *m* = 1 vortex solitons with different propagation constants are compared. While in $C_{4}$ lattice, only vortex solitons with *m* = 1 were obtained, in $C_{5}$ lattices, we found *m* = 1 [Figure 3(b)] and *m* = 2 [Figure 3(c)] families (for properties of higher-charge nonlinear states in $C_{7,8}$ lattices see [72]). Field modulus and phase distributions for the latter states are presented below corresponding *U*(*β*) distributions. Notice that in a nontopological lattice achieved by setting *γ* < 1, no linear localized states are sustained within the gap; thus, vortex solitons can only appear above some power threshold.

One of the most essential aspects for potential experimental realization is the stability of disclination vortex solitons and this is where they show properties strongly departing from properties of vortex solitons in nontopological lattices. A linear stability analysis and modeling of propagation were performed for the perturbed vortex solitons. We searched for perturbed solutions of Eq. (1) in the form $\Psi \left(x,y,z\right)=\left[\phi_{r}\left(x,y\right)+i\phi_{i}\left(x,y\right)+\right.\left.\;u\left(x,y\right)e^{\delta z}+iv\left(x,y\right)e^{\delta z}\right]e^{i\beta z}$ , where *u* and *v* are real and imaginary parts of perturbation, respectively. Linearization of Eq. (1) around *ϕ*_*r*_ and *ϕ*_*i*_ yields the eigenvalue problem:

(3) $$\begin{array}{c} \delta u=\left[-\frac{1}{2}\nabla^{2}+\beta -g\left(\phi_{r}^{2}+3\phi_{i}^{2}\right)-V\right]v-2g\phi_{r}\phi_{i}u,\\ \delta v=\left[+\frac{1}{2}\nabla^{2}-\beta +g\left(3\phi_{r}^{2}+\phi_{i}^{2}\right)+V\right]u+2g\phi_{r}\phi_{i}v, \end{array}$$

which was solved to obtain a perturbation growth rate for all possible perturbations *δ* = *δ*_re_ + *iδ*_im_. Vortex solitons are stable when *δ*_re_ = 0. In Figure 3, stable branches are shown in black, while unstable ones are shown in red. Vortex solitons with *m* = 1 supported by $C_{4}$ lattices (according to the group theory arguments, this is the only possible charge of vortex soliton in the lattice with this discrete rotational symmetry) are stable in the entire range of their existence, in both focusing and defocusing media, as shown in Figure 3(a). In $C_{5}$ lattice (where only compact vortex states with topological charges up to *m* = 2 are allowed on the disclination core), *m* = 1 vortex solitons are stable [Figure 3(b)], while *m* = 2 ones are unstable [Figure 3(c)] in focusing medium (*g* = +1). Moreover, stability properties in $C_{5}$ lattice change completely when the nonlinearity sign changes (*g* = −1), so that the families that were unstable in the focusing medium become stable in defocusing one, and vice versa [74]. It should be stressed that different stability properties of vortex solitons with lower and higher charges are typical for lattices with discrete rotational symmetry, but, at the same time, stability properties reported here are in clear contrast to those found in nontopological lattices [19], [21], where, for example, in focusing medium only solitons with highest topological charges can be stable. We believe that this difference is connected with the fact that topological disclination vortex solitons appear in finite topological gap and on this reason their formation mechanism is different from that of conventional vortex solitons in semi-infinite gap. Although vortex solitons in disclination lattices predominantly occupy the central sites within the disclination, they are fundamentally distinct from vortex solitons in nontopological ring-like waveguide arrangements, i.e., the structures obtained by excluding all waveguides that are not part of the disclination core [75], as their stability characteristics are completely different from each other [72].

**Figure 4:**
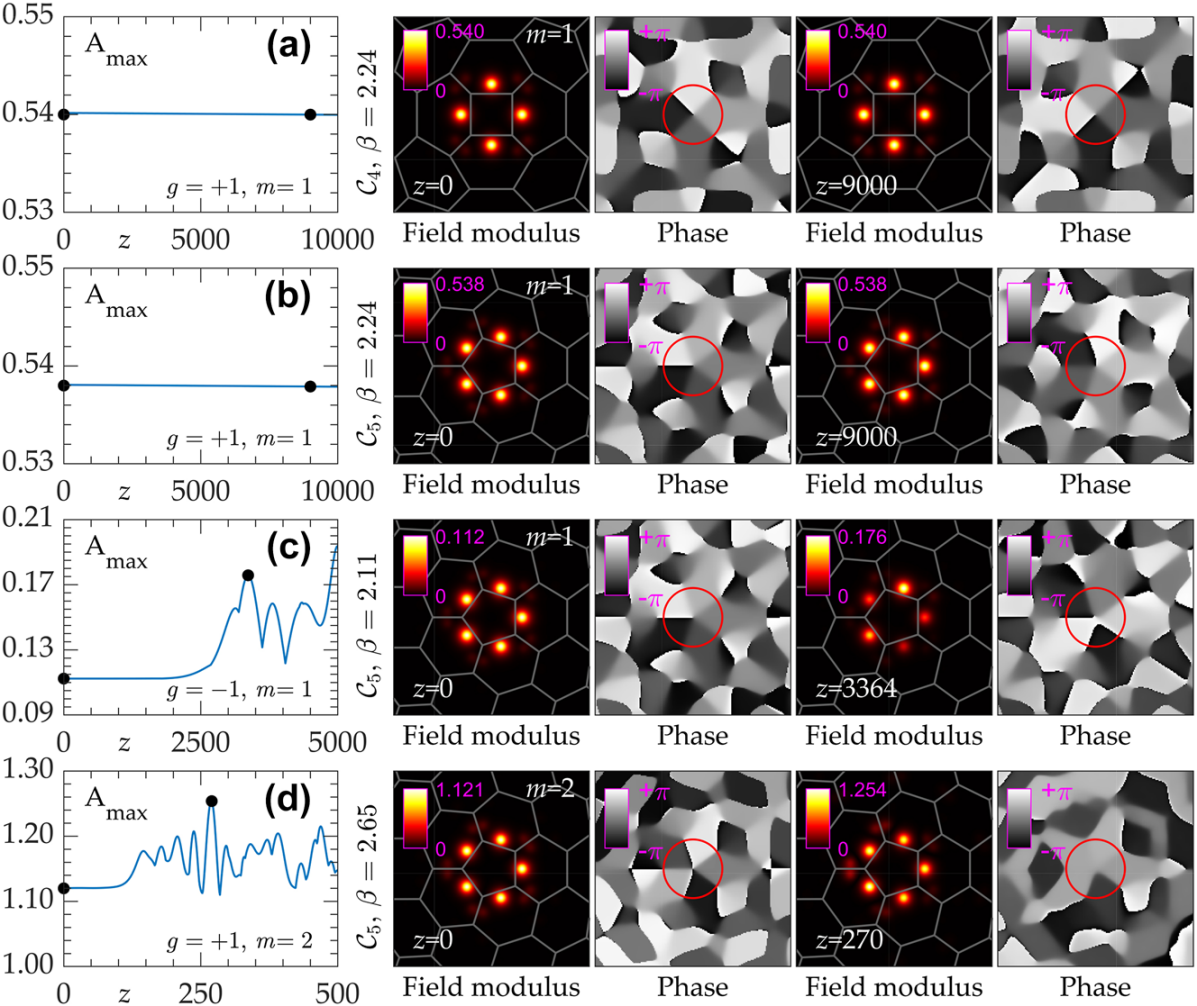
Propagation dynamics of stable (a), (b) and unstable (c), (d) vortex solitons. Peak amplitude *A*_max_ versus propagation distance *z* is shown in the left plots, while field modulus and phase distributions corresponding to the black dots on *A*_max_(*z*) plots are displayed in the right panels. (a) $C_{4}$ lattice, *β* = 2.24, *m* = 1, (b) $C_{5}$ lattice, *β* = 2.24, *m* = 1, (c) $C_{5}$ lattice, *β* = 2.11, *m* = 1, and (d) $C_{5}$ lattice, *β* = 2.65, *m* = 2 in (d). In (a), (b), and (d) *g* = +1, while in (c) *g* = −1.

Figure 4(a) and (b) show examples of long-range stable propagation of *m* = 1 vortex solitons in $C_{4}$ and $C_{5}$ disclination lattices, with dependencies of peak amplitude *A*_max_ = max |Ψ| on distance *z* and snapshots at different distances, obtained by direct numerical integration of Eq. (1) using split-step fast Fourier method. Field modulus distributions in such states remain undistorted even after *z* ∼ 10^4^. At the same time, unstable *m* = 1 states in defocusing medium [Figure 4(c)] and unstable *m* = 2 states in focusing medium [Figure 4(d)] in $C_{5}$ lattice show growing amplitude oscillations and typically lose vortical phase structure. The development of instability results in intensity oscillations of the spots, contributing significantly to the emission of radiation into the bulk of the lattice. This highlights the significance of the array surrounding the disclination core in defining the vortex soliton, distinct from the ring-like structures. Examples of stable and unstable evolution of vortices in $C_{7,8}$ lattices are presented in [72].

## 5 Conclusion and outlook

We have shown that the symmetry of the disclination lattice plays a crucial role in the formation of vortex solitons of topological origin in the spectral topological gap. The formation of such states is facilitated by discrete rotational symmetry of the disclination lattice that simultaneously imposes strict restrictions on the topological charges of symmetric vortex solitons and determines their stability properties. In some cases, the symmetry can protect solitons from perturbations that would otherwise cause their decay, leading to unusual stability properties. Studying the interplay between nonlinearity and topology is essential for the development of new materials with desirable properties and the exploration of new opportunities for all-optical control of topological excitations, especially when they possess an orbital degree of freedom.

## Supplementary Material

Supplementary Material Details

## References

[1] Desyatnikov A. S., Kivshar Y. S., Torner L. (2005). Optical vortices and vortex solitons. *Prog. Opt.*.

[2] Kartashov Y. V., Astrakharchik G., Malomed B. A., Torner L. (2019). Frontiers in multidimensional self-trapping of nonlinear fields and matter. *Nat. Rev. Phys.*.

[3] Malomed B. A. (2019). Vortex solitons: old results and new perspectives. *Phys. D*.

[4] Mihalache D. (2021). Localized structures in optical and matter-wave media: a selection of recent studies. *Rom. Rep. Phys.*.

[5] Pryamikov A., Hadzievski L., Fedoruk M., Turitsyn S., Aceves A. (2021). Optical vortices in waveguides with discrete and continuous rotational symmetry. *J. Eur. Opt. Soc.-Rapid Publ.*.

[6] Kevrekidis P. G., Frantzeskakis D. J., Carretero-González R. (2008). Emergent nonlinear phenomena in Bose–Einstein condensates. *Springer Ser. At., Opt., Plasma Phys.*.

[7] Fetter A. L. (2010). Vortices and dynamics in trapped Bose–Einstein condensates. *J. Low Temp. Phys.*.

[8] Kevrekidis P. G., Frantzeskakis D. J., Carretero-González R. (2015). *The Defocusing Nonlinear Schrödinger Equation: From Dark Solitons to Vortices and Vortex Rings*.

[9] Carusotto I., Ciuti C. (2013). Quantum fluids of light. *Rev. Mod. Phys.*.

[10] Padgett M., Bowman R. (2011). Tweezers with a twist. *Nat. Photonics*.

[11] Zhang Z. (2020). Tunable topological charge vortex microlaser. *Science*.

[12] Torner L., Torres J., Carrasco S. (2005). Digital spiral imaging. *Opt. Express*.

[13] Molina-Terriza G., Torres J. P., Torner L. (2007). Twisted photons. *Nat. Phys.*.

[14] Kavokin A., Liew T. C. H., Schneider C., Lagoudakis P. G., Klembt S., Hoefling S. (2022). Polariton condensates for classical and quantum computing. *Nat. Rev. Phys.*.

[15] Malomed B. A., Kevrekidis P. G. (2001). Discrete vortex solitons. *Phys. Rev. E*.

[16] Yang J., Musslimani Z. H. (2003). Fundamental and vortex solitons in a two-dimensional optical lattice. *Opt. Lett.*.

[17] Neshev D. N. (2004). Observation of discrete vortex solitons in optically induced photonic lattices. *Phys. Rev. Lett.*.

[18] Fleischer J. W. (2004). Observation of vortex-ring discrete solitons in 2d photonic lattices. *Phys. Rev. Lett.*.

[19] Law K. J. H., Kevrekidis P. G., Alexander T. J., Krolikowski W., Kivshar Y. S. (2009). Stable higher-charge discrete vortices in hexagonal optical lattices. *Phys. Rev. A*.

[20] Ferrando A., Zacares M., Garcia-March M. A. (2005). Vorticity cutoff in nonlinear photonic crystals. *Phys. Rev. Lett.*.

[21] Kartashov Y. V., Ferrando A., Egorov A. A., Torner L. (2005). Soliton topology versus discrete symmetry in optical lattices. *Phys. Rev. Lett.*.

[22] Dong L., Kartashov Y. V., Torner L., Ferrando A. (2022). Vortex solitons in twisted circular waveguide arrays. *Phys. Rev. Lett.*.

[23] Poddubny A. N., Smirnova D. A. (2018). Ring Dirac solitons in nonlinear topological systems. *Phys. Rev. A*.

[24] Li R. (2022). Topological bulk solitons in a nonlinear photonic chern insulator. *Commun. Phys.*.

[25] Nedić M., Gligorić G., Petrovic J., Maluckov A. (2023). Nonlinearity and lasing topological zero-mode in distorted photonic lattice. *Phys. Lett. A*.

[26] Zak J. (1989). Berry’s phase for energy bands in solids. *Phys. Rev. Lett.*.

[27] Vanderbilt D., King-Smith R. D. (1993). Electric polarization as a bulk quantity and its relation to surface charge. *Phys. Rev. B*.

[28] Hasan M. Z., Kane C. L. (2010). Colloquium: topological insulators. *Rev. Mod. Phys.*.

[29] Qi X.-L., Zhang S.-C. (2011). Topological insulators and superconductors. *Rev. Mod. Phys.*.

[30] Benalcazar W. A., Bernevig B. A., Hughes T. L. (2017). Quantized electric multipole insulators. *Science*.

[31] Benalcazar W. A., Bernevig B. A., Hughes T. L. (2017). Electric multipole moments, topological multipole moment pumping, and chiral hinge states in crystalline insulators. *Phys. Rev. B*.

[32] Schindler F. (2018). Higher-order topology in bismuth. *Nat. Phys.*.

[33] Peterson C. W., Benalcazar W. A., Hughes T. L., Bahl G. (2018). A quantized microwave quadrupole insulator with topologically protected corner states. *Nature*.

[34] Xue H., Yang Y., Gao F., Chong Y., Zhang B. (2018). Acoustic higher-order topological insulator on a Kagome lattice. *Nat. Mat.*.

[35] Mittal S., Orre V. V., Zhu G., Gorlach M. A., Poddubny A., Hafezi M. (2019). Photonic quadrupole topological phases. *Nat. Photon.*.

[36] Khalaf E., Benalcazar W. A., Hughes T. L., Queiroz R. (2021). Boundary-obstructed topological phases. *Phys. Rev. Res.*.

[37] Xie B. (2021). Higher-order band topology. *Nat. Rev. Phys.*.

[38] Lumer Y., Plotnik Y., Rechtsman M. C., Segev M. (2013). Self-localized states in photonic topological insulators. *Phys. Rev. Lett.*.

[39] Ablowitz M. J., Curtis C. W., Ma Y. P. (2014). Linear and non-linear traveling edge waves in optical honeycomb lattices. *Phys. Rev. A*.

[40] Leykam D., Chong Y. D. (2016). Edge solitons in nonlinear-photonic topological insulators. *Phys. Rev. Lett.*.

[41] Kartashov Y. V., Skryabin D. V. (2016). Modulational instability and solitary waves in polariton topological insulators. *Optica*.

[42] Li C. (2018). Lieb polariton topological insulators. *Phys. Rev. B*.

[43] Mukherjee S., Rechtsman M. C. (2020). Observation of floquet solitons in a topological bandgap. *Science*.

[44] Maczewsky L. J. (2020). Nonlinearity-induced photonic topological insulator. *Science*.

[45] Xia S. Q. (2020). Nontrivial coupling of light into a defect: the interplay of nonlinearity and topology. *Light Sci. Appl.*.

[46] Mukherjee S., Rechtsman M. C. (2021). Observation of unidirectional soliton-like edge states in nonlinear floquet topological insulators. *Phys. Rev. X*.

[47] Kirsch M. S. (2021). Nonlinear second-order photonic topological insulators. *Nat. Phys.*.

[48] Hu Z. C. (2021). Nonlinear control of photonic higher-order topological bound states in the continuum. *Light Sci. Appl.*.

[49] Zangeneh-Nejad F., Fleury R. (2019). Nonlinear second-order topological insulators. *Phys. Rev. Lett.*.

[50] Smirnova D. A., Smirnov L. A., Leykam D., Kivshar Y. S. (2019). Topological edge states and gap solitons in the nonlinear Dirac model. *Las. Photon. Rev.*.

[51] Kartashov Y. V. (2022). Observation of edge solitons in topological trimer arrays. *Phys. Rev. Lett.*.

[52] Bahari B., Ndao A., Vallini F., Amili A. E., Fainman Y., Kanté B. (2017). Nonreciprocal lasing in topological cavities of arbitrary geometries. *Science*.

[53] Harari G. (2018). Topological insulator laser: theory. *Science*.

[54] Bandres M. A. (2018). Topological insulator laser: experiments. *Science*.

[55] Kartashov Y. V., Skryabin D. V. (2019). Two-dimensional topological polariton laser. *Phys. Rev. Lett.*.

[56] Kruk S. S., Gao W., Choi D. Y., Zentgraf T., Zhang S., Kivshar Y. (2021). Nonlinear imaging of nanoscale topological corner states. *Nano Lett.*.

[57] Jürgensen M., Mukherjee S., Rechtsman M. C. (2021). Quantized nonlinear thouless pumping. *Nature*.

[58] Fu Q., Wang P., Kartashov Y. V., Konotop V. V., Ye F. (2022). Nonlinear thouless pumping: solitons and transport breakdown. *Phys. Rev. Lett.*.

[59] Jürgensen M., Rechtsman M. C. (2022). Chern number governs soliton motion in nonlinear thouless pumps. *Phys. Rev. Lett.*.

[60] Fu Q., Wang P., Kartashov Y. V., Konotop V. V., Ye F. (2022). Two-dimensional nonlinear thouless pumping of matter waves. *Phys. Rev. Lett.*.

[61] Jürgensen M., Mukherjee S., Jörg C., Rechtsman M. C. (2023). Quantized fractional thouless pumping of solitons. *Nat. Phys.*.

[62] Teo J. C., Hughes T. L. (2013). Existence of majorana-fermion bound states on disclinations and the classification of topological crystalline superconductors in two dimensions. *Phys. Rev. Lett.*.

[63] Benalcazar W. A., Teo J. C., Hughes T. L. (2014). Classification of two-dimensional topological crystalline superconductors and majorana bound states at disclinations. *Phys. Rev. B*.

[64] Li T., Zhu P., Benalcazar W. A., Hughes T. L. (2020). Fractional disclination charge in two-dimensional *C*
_n_-symmetric topological crystalline insulators. *Phys. Rev. B*.

[65] Peterson C. W., Li T., Jiang W., Hughes T. L., Bahl G. (2021). Trapped fractional charges at bulk defects in topological insulators. *Nature*.

[66] Liu Y. (2021). Bulk-disclination correspondence in topological crystalline insulators. *Nature*.

[67] Wu S., Jiang B., Liu Y., Jiang J.-H. (2021). All-dielectric photonic crystal with unconventional higher-order topology. *Photon. Res.*.

[68] Hwang M.-S., Kim H.-R., Kim J., Yang B.-J., Kivshar Y., Park H.-G. (2023). Vortex nanolaser based on a photonic disclination cavity. *Nat. Photonics*.

[69] Wang Q., Xue H., Zhang B., Chong Y. D. (2020). Observation of protected photonic edge states induced by real-space topological lattice defects. *Phys. Rev. Lett.*.

[70] Chen Y. (2022). Observation of topological p-orbital disclination states in non-euclidean acoustic metamaterials. *Phys. Rev. Lett.*.

[71] Deng Y., Benalcazar W. A., Chen Z.-G., Oudich M., Ma G., Jing Y. (2022). Observation of degenerate zero-energy topological states at disclinations in an acoustic lattice. *Phys. Rev. Lett.*.

[72] Supplementary Material contains information on linear spectra of 
$C_{7,8}$
 lattices and corresponding vortex soliton families existing in such structures. It also provides the stability scenario for vortex solitons in ring-like structures. ..

[73] Ren B., Wang H., Kartashov Y. V., Li Y., Zhang Y. (2023). Nonlinear photonic disclination states. *APL Photonics*.

[74] Kevrekidis P., Susanto H., Chen Z. (2006). High-order-mode soliton structures in two-dimensional lattices with defocusing nonlinearity. *Phys. Rev. E*.

[75] Desyatnikov A. S., Dennis M. R., Ferrando A. (2011). All-optical discrete vortex switch. *Phys. Rev. A*.

