# Supplementary material for: Vortex solitons in topological disclination lattices
Source: Nanophotonics. 2024 Jan 22;13(18):3495–502. doi: 10.1515/nanoph-2023-0790 (PMC11501678; doi:10.1515/nanoph-2023-0790)
Supplement: Supplementary file 1 — Supplementary Material Details [file j_nanoph-2023-0790_suppl_001.pdf]

Supplementary Material for
Vortex solitons in topological disclination lattices

Changming Huang,[1] Ce Shang,[2,*] Yaroslav V. Kartashov,[3] and Fangwei Ye[4,†]

[1] *Department of Physics, Changzhi University, Changzhi, Shanxi 046011, China*
[2] *King Abdullah University of Science and Technology (KAUST), Physical Science and Engineering Division (PSE), Thuwal 23955-6900, Saudi Arabia.*
[3] *Institute of Spectroscopy, Russian Academy of Sciences, 108840, Troitsk, Moscow, Russia*
[4] *School of Physics and Astronomy, Shanghai Jiao Tong University, Shanghai 200240, China*

(Dated: December 27, 2023)

In this Supplementary Material, we discuss the topological characterization of the disclination lattices and provide the results for linear spectra and vortex solitons in disclination lattices with higher, $\mathcal{C}_7$ and $\mathcal{C}_8$, discrete rotational symmetry. Such lattices are shown to support vortex solitons with topological charges up to $m = \pm 3$. The stability properties of such vortex solitons and the effect of the intrinsic loss of the waveguide array on their stability are discussed. Additionally, we conduct a systematic study comparing the stability of vortex solitons in a ring of waveguides with stability of vortex solitons supported by the disclination lattices, aiming to highlight the significance of the surrounding waveguide array in the stability of vortex solitons in disclination lattices.

1, DISCLINATION LATTICES WITH $\mathcal{C}_7$ AND $\mathcal{C}_8$ SYMMETRY

To create disclination lattices with discrete rotational symmetry higher than $\mathcal{C}_6$, we insert into the original hexagonal sample [Fig. S1(a)] the $n\pi/3$ sector, as shown in Fig. S1. We compress the cells accordingly so that the lattice accommodates the newly added sector. Specifically, when $n = 1$, the resulting lattice exhibits $\mathcal{C}_7$ discrete rotational symmetry [Fig. S1(b)], while for $n = 2$ the $\mathcal{C}_8$ structure can be obtained [Fig. S1(c)]. The declination core is clearly visible in the center of each such structure.

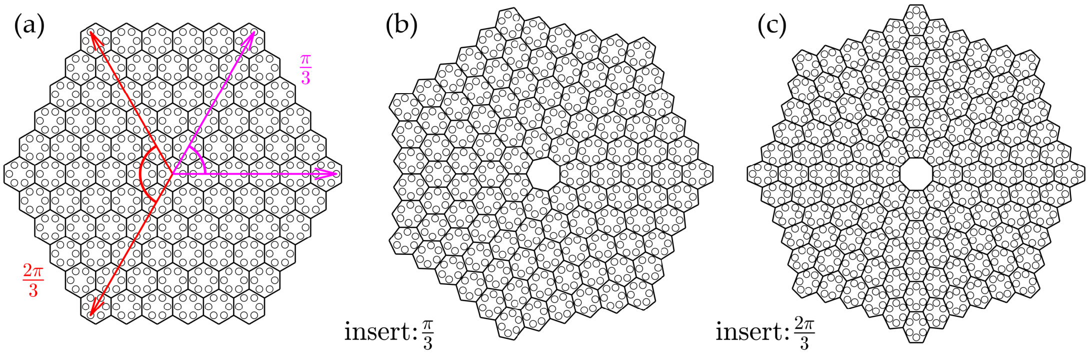

FIG. S1. Schematic illustration of the method of construction of the disclination lattices with higher discrete rotational symmetry. (a) Original hexagonal lattice structure. (b),(c) Disclination lattices with $\mathcal{C}_7$ (or $\mathcal{C}_8$) symmetry can be generated after insertion of a $\pi/3$ (or $2\pi/3$) sector into the hexagonal structure and subsequent compression of lattice cells.

2, LINEAR MODES IN DISCLINATION LATTICES WITH $\mathcal{C}_7$ AND $\mathcal{C}_8$ SYMMETRY

The increase of the order of discrete rotational symmetry N leads to an increase in the number of linear modes localized at the disclination core of the $\mathcal{C}_N$ lattice. This phenomenon offers an opportunity for the generation of vortex states with higher topological charges. Therefore, it is essential to investigate the properties of linear localized disclination modes in $\mathcal{C}_7$ and $\mathcal{C}_8$ lattices.

These results once again confirm the conclusion drawn in the main text that the available charge of disclination vortex in $\mathcal{C}_N$ lattice is given by $m < N/2$ (for even N) and $m < (N+1)/2$ (for odd N). It is interesting to note that degenerate linear modes of disclination lattices that one can use for the construction of vortex states typically feature

* shang.ce@kaust.edu.sa
† fangweiye@sjtu.edu.cn

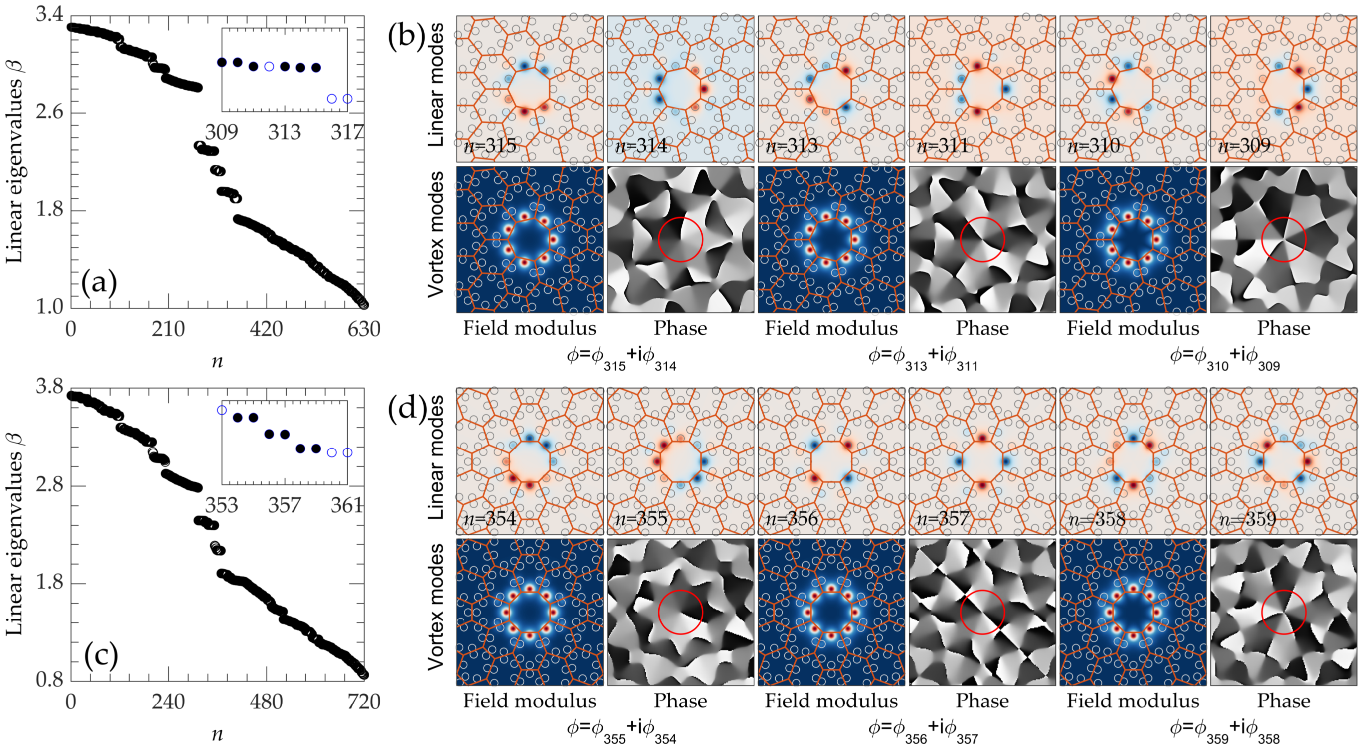

FIG. S2. Linear eigenvalues, examples of linear disclination modes, and vortex modes that can be obtained in lattices with $\mathcal{C}_7$ and $\mathcal{C}_8$ symmetry. Linear eigenvalues of modes supported by the $\mathcal{C}_7$ (a) and $\mathcal{C}_8$ (c) lattice at $\gamma = 1.72$ are shown. Solid dots in the insets indicate pairs of degenerate disclination modes that can be used for the construction of vortex states. The profiles of three pairs of degenerate eigenmodes supported by the $\mathcal{C}_7$ (b) or $\mathcal{C}_8$ (d) symmetric lattices (top row) and the corresponding vortex modes they generate (bottom row). In $\mathcal{C}_7$ lattice the degenerate pairs of disclination states are $\phi_{n=315,314}$, $\phi_{n=313,311}$, and $\phi_{n=310,309}$. In $\mathcal{C}_8$ lattice the degenerate pairs of disclination states are $\phi_{n=354,355}$, $\phi_{n=356,357}$, and $\phi_{n=358,359}$. The organge lines in (b) and (d) depict unit cells, while circles indicate the positions of the waveguides.

opposite symmetries with respect to some axis in the (x, y) plane [for instance, $\phi_{n=315}$ and $\phi_{n=314}$ modes from Fig. S2(b) are anti-symmetric (symmetric) with respect to $y = 0$ axis].

3, VORTEX SOLITONS IN DISCLINATION LATTICES WITH $\mathcal{C}_7$ AND $\mathcal{C}_8$ SYMMETRY

Vortex solitons with different topological charges bifurcating from linear topological vortex states have been found in both $\mathcal{C}_7$ and $\mathcal{C}_8$ lattices (see Fig. S3). The $U(\beta)$ dependencies for these vortex solitons are qualitatively similar to those in $\mathcal{C}_4$ and $\mathcal{C}_5$ lattices. One can observe that nonlinear vortex states bifurcate from linear ones in both focusing and defocusing medium, with power gradually increasing toward the edge of the gap. The 7-peak and 8-peak vortex solitons are well localized around the disclination core, their vortical phase distributions with phase singularity in the center are highlighted by the red circles in phase structures shown in Fig. S3.

We present a quantitative analysis of the light power confinement around the disclination core, denoted as U_c and defined as $U_c = \int_0^{2\pi} \int_{r_0-w}^{r_0+w} |\phi|^2 r dr d\theta$. Here, r_0 represents the radius measured from the disclination core to the center of the central waveguides of the disclination lattices, w is the waveguide width, and θ represents the angle in polar coordinates. The light power confinement U_c, and the ratio of the light power confinement around the disclination core to total power of the vortex soliton (U_c/U) are depicted in Figs. S4(a), S4(b), S4(c), and S4(d). We observe that the light power confinement around the disclination core, U_c, exhibits the same trend of variation with β as the $U(\beta)$ curve, while the fraction of power localized on disclination core U_c/U, typically demonstrates an opposite variation with the strength of the nonlinearity in focusing nonlinearity compared to in defocusing nonlinearity. However, throughout their entire existence region, the fraction of power localized on disclination core remains above 60%, indicating their nature as nonlinear states around the core of the disclination lattices.

The stability picture for solitons in $\mathcal{C}_7$ and $\mathcal{C}_8$ lattices is more complex, which we determined through rigorous linear stability analysis. In Fig. S3 stable and unstable branches are shown with black and red colors, respectively, in corresponding $U(\beta)$ curves. It is interesting that stable $m = 1$ vortex solitons have been found in $\mathcal{C}_7$ lattice in focusing medium, while in $\mathcal{C}_8$ lattice such solitons are already unstable for $g = +1$. As before, stability properties for

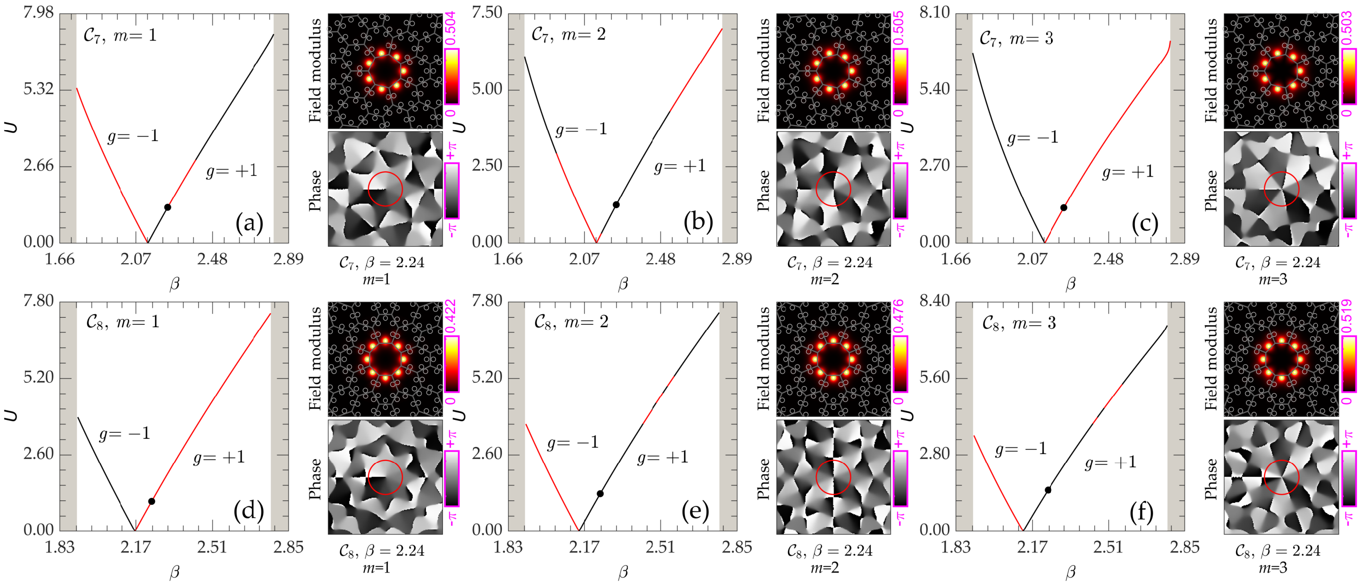

FIG. S3. Vortex solitons supported by $\mathcal{C}_7$ and $\mathcal{C}_8$ symmetric lattices. The $U(\beta)$ curves for vortex gap solitons with $m = 1$, $\mathcal{C}_7$ lattice (a), $m = 2$, $\mathcal{C}_7$ lattice (b), $m = 3$, $\mathcal{C}_7$ lattice (c), $m = 1$, $\mathcal{C}_8$ lattice (d), $m = 2$, $\mathcal{C}_8$ lattice (e), $m = 3$, $\mathcal{C}_8$ lattice (f), are plotted. The gray regions in the $U(\beta)$ plots illustrate bulk bands. Stable families are indicated by black lines, while unstable families are indicated by red lines. Examples of vortex solitons are attached to the side of their respective $U(\beta)$ panels. The gray lines in the field modulus distributions depict the lattice unit cells, and the circles correspond to the position of the waveguides. Phase singularity in the center of each pattern is highlighted by the red circle.

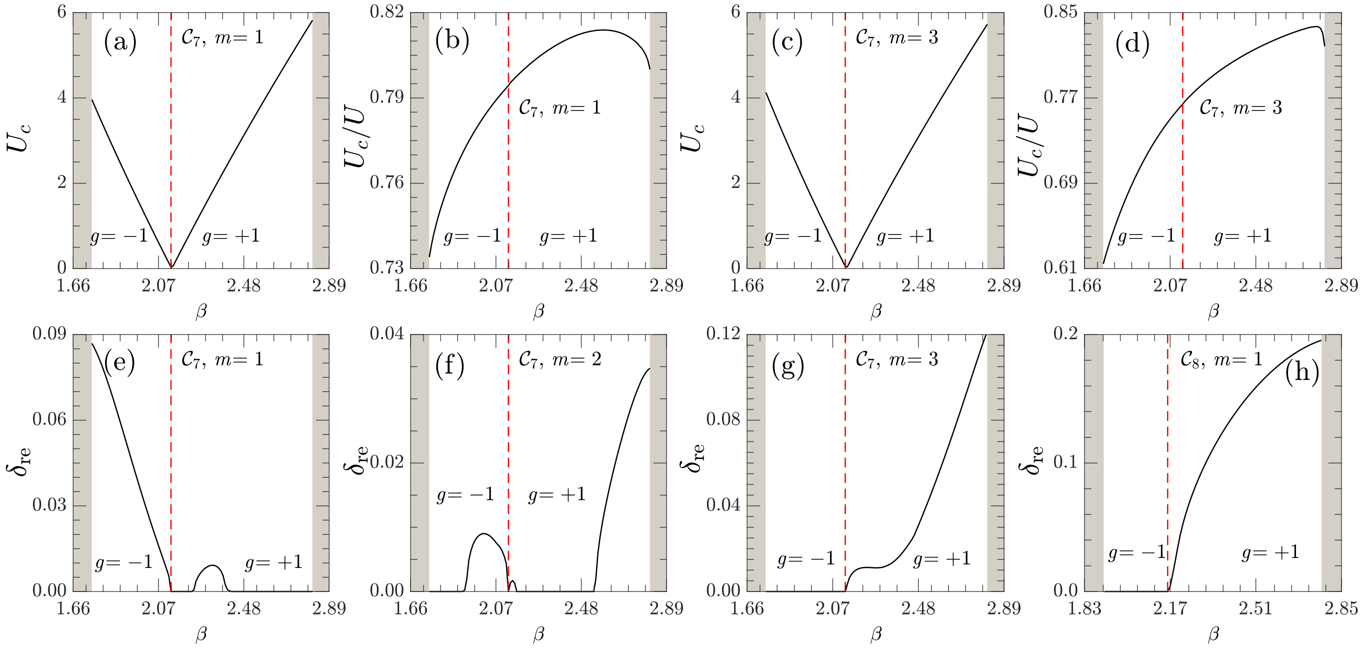

FIG. S4. The dependence of light power concentrated around the disclination core U_c (or U_c/U)(the first line), and the dependence of real part of the perturbation growth rate $\delta_{\rm re}$ (the second line), on the propagation constant β. The $U_c(\beta)$ and $U_c/U(\beta)$ curves for vortex solitons with $m = 1$, $\mathcal{C}_7$ lattice (a,b), $m = 3$, $\mathcal{C}_7$ lattice (c,d), are plotted. The $\delta_{\rm re}(\beta)$ curves for vortex solitons with $m = 1$, $\mathcal{C}_7$ lattice (e), $m = 2$, $\mathcal{C}_7$ lattice (f), $m = 3$, $\mathcal{C}_7$ lattice (g), $m = 1$, $\mathcal{C}_8$ lattice (h), are plotted. The red dashed line represents the boundary between focusing and defocusing nonlinearities.

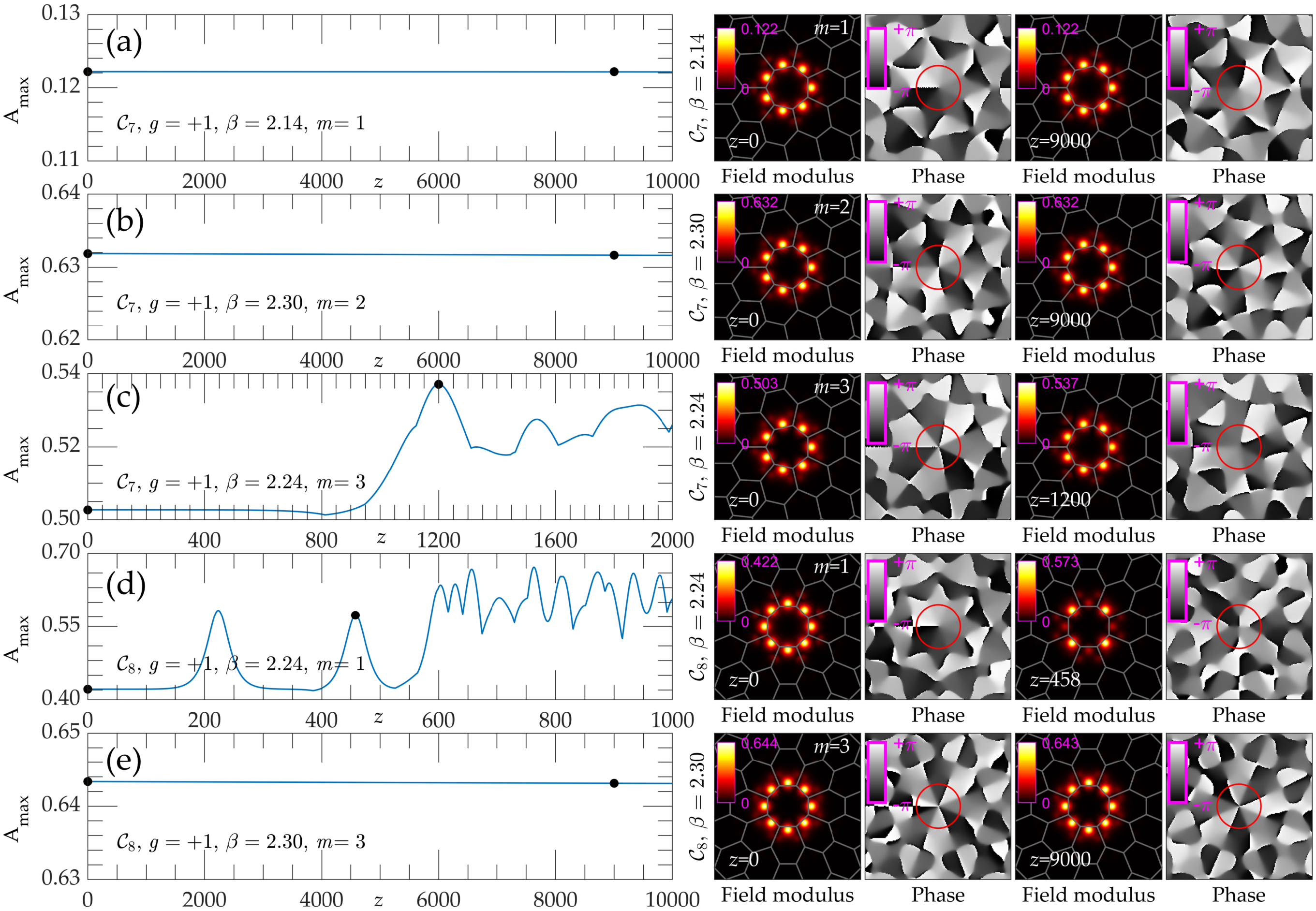

FIG. S5. Propagation dynamics of vortex gap solitons in $\mathcal{C}_7$ and $\mathcal{C}_8$ symmetric lattices. The maximum amplitude $A_{\max}$ of the field versus the propagation distance z is shown on the left plots, while snapshots with field modulus and phase distributions corresponding to the black dots in the $A_{\max}(z)$ plots are presented on the right. The evolution of stable vortex solitons is shown in panels (a), (b), and (c), while the unstable evolution is shown in panels (c) and (d). The gray lines in the field modulus panel depict the lattice cells, and the circles correspond to the position of the waveguides. $m=1$, $\mathcal{C}_7$ lattice, $\beta=2.14$ in (a), $m=2$, $\mathcal{C}_7$ lattice, $\beta=2.3$ in (b), $m=3$, $\mathcal{C}_7$ lattice, $\beta=2.24$ in (c), $m=1$, $\mathcal{C}_8$ lattice, $\beta=2.24$ in (d), and $m=3$, $\mathcal{C}_8$ lattice, $\beta=2.3$ in (e).

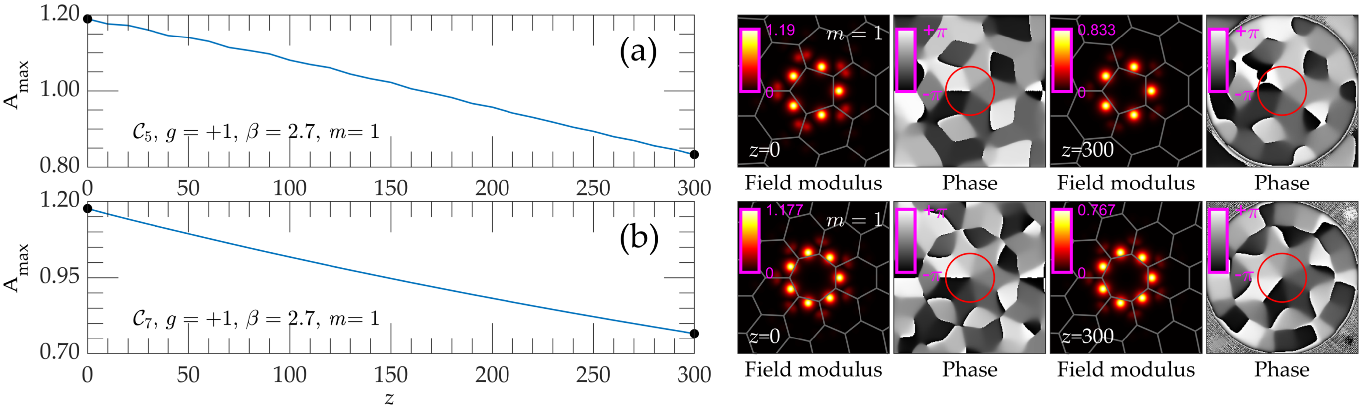

FIG. S6. Examples of the propagation dynamics of vortex solitons with an absorption coefficient $\alpha=0.00131$. The maximum amplitude $A_{\max}$ of the field versus the propagation distance z is shown on the left plots, while snapshots with field modulus and phase distributions corresponding to the black dots in the $A_{\max}(z)$ plots are presented on the right. The gray lines in the field modulus panel depict the lattice cells, and the circles correspond to the position of the waveguides. $m=1$, $\mathcal{C}_5$ lattice, $\beta=2.7$ in (a), $m=1$, $\mathcal{C}_7$ lattice, $\beta=2.7$ in (b).

states with propagation constants not too far from the propagation constant of linear mode change when the sign of nonlinearity g changes. Vortex solitons with $m = 2$ and $m = 3$ charges may have complex stability domains in $\mathcal{C}_8$ structure and for $g = +1$.

The examples of stable propagation of vortex solitons with different topological charges, which maintain their field modulus and phase distributions even after a considerable propagation distance ($z \sim 10000$) in $\mathcal{C}_7$ and $\mathcal{C}_8$ disclination lattices are shown in Figs. S5(a), S5(b), and S5(e). Unstable vortex solitons exhibit two distinct types of behavior: (1) the field modulus pattern maintains its shape but with small amplitude fluctuations, while the phase structure is lost after propagation over a certain distance [see Fig. S5(c)]; (2) bright spots in vortex profile show strong oscillations and after some propagation distance the field modulus distribution changes dramatically (the number of spots decreases) and vortical phase structure is lost as well [see Fig. S5(d)].

To further demonstrate that the vortex solitons evolve stably over experimentally feasible distances, we take into account the loss of waveguides. Such losses ~ 0.1dB/cm can be taken into account by including the term $-i\alpha\Psi$ into the right-hand side of Eq. (1), where the absorption coefficient is as small as $\alpha \approx 0.00131$. The corresponding results are shown in Fig. S6. One can observe that the amplitude of the input vortex soliton gradually decreases with the propagation distance. However, within the range of an experimentally feasible sample length ($z \approx 175 \leftrightarrow 20$ cm), the field modulus distribution changes only slightly (mainly due to decrease of peak amplitude of soliton) and representative phase structure is clearly conserved.

It is interesting and relevant to consider the effect of perturbations that break the exact $\mathcal{C}_N$ discrete rotational symmetry of a structure on the evolution of vortex modes. To analyze the effects of these perturbations, we introduced disorder into all waveguides of the structure, including those located at the disclination core, by allowing their depths to change randomly within the interval $[p - \delta, p + \delta]$, where the disorder level δ is much smaller than p. We then propagated the linear vortex modes obtained in the unperturbed lattice with $\mathcal{C}_N$ symmetry in the lattice with the disorder, which does not possess any discrete rotational symmetry. The results are shown in Fig. S7, with the disorder level $\delta = 0.05p$. In all cases, we observed that the vortex modes with $m = 1$ persist throughout the evolution [Fig. S7 (a)]. However, vortices with higher charges may exhibit splitting of the singularity at the center. This is clearly seen in Fig. S7 (b) and (c), where the vortex with $m = 2$ quickly splits its double-folded singularity into two single

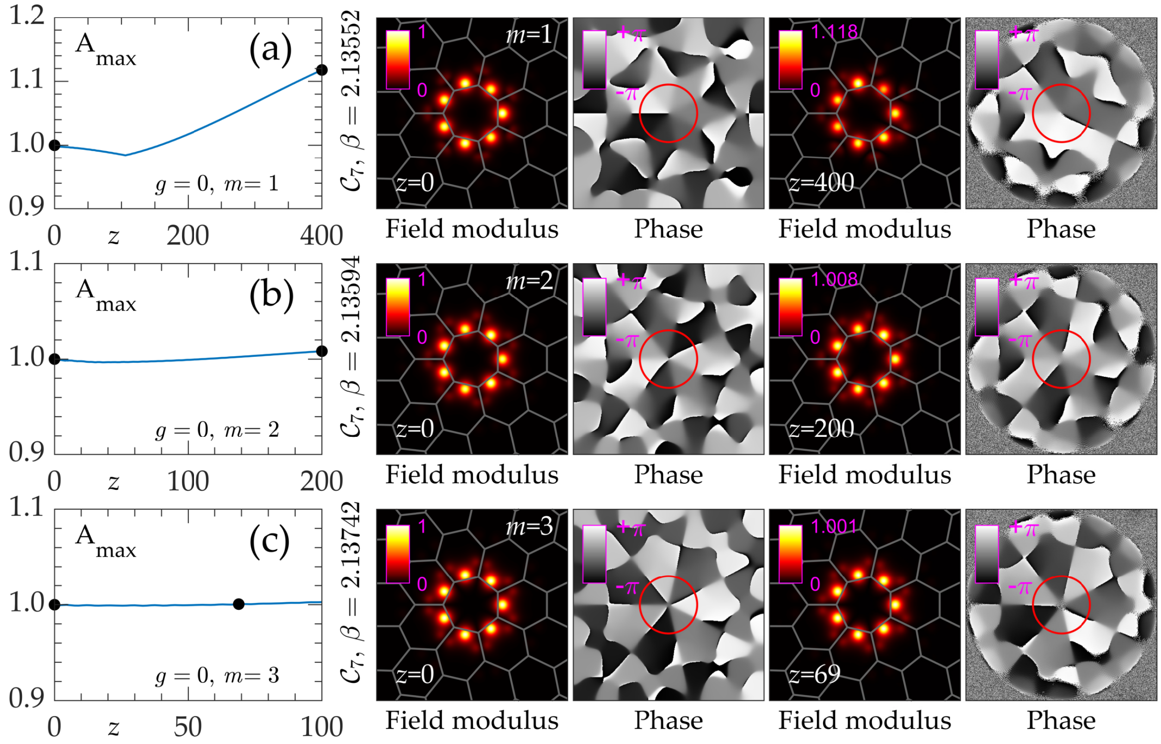

FIG. S7. Propagation dynamics of linear vortex modes with $m = 1, 2, 3$ in 5% level noise perturbed $\mathcal{C}_7$ symmetric lattices. The maximum amplitude $A_{\max}$ of the field versus the propagation distance z is shown on the left panels, while the snapshots with field modulus and phase distributions corresponding to the black dots in the $A_{\max}(z)$ plots are presented on the right panels. The gray lines in the field modulus panel depict the lattice cells, and the circles correspond to the position of the waveguides. $m = 1$, $\beta = 2.13552$ in (a), $m = 2$, $\beta = 2.13594$ in (b), and $m = 3$, $\beta = 2.13742$ in (c).

singularities, and the vortex with $m = 3$ splits its triple-folded singularity into three single singularities. Nevertheless, the removal of this degeneracy in phase singularity does not result in the destruction of the vortex state, as evidently shown in all the propagation simulations of Fig. S7.

4, VORTEX SOLITONS IN RING-LIKE LATTICES

In this section, we conduct a systematic study on the stability of vortex solitons in a ring of waveguides and compare it to that of vortex solitons in disclination lattices. To create the ring-like waveguide, we remove all waveguides from the disclination lattices that are not part of the disclination core, as shown in Fig. S8. Our study considers both focusing and defocusing nonlinearities.

We observe two key differences between vortex solitons in ring-like waveguides and those in disclination lattices. In the ring-like waveguides, as shown in Fig. S8, the domain of existence for vortex solitons is much broader compared to that of gap vortex solitons in topological disclination lattices. This is because the ring-like structure lacks band structures that could determine the existence domain of the vortex solitons. Additionally, the power of these vortex solitons is entirely confined within the ring-like waveguide structure. As a result, the vortex solitons in the ring-like waveguides always have monotonically decaying tails, which is distinct from their counterparts in disclination lattices where the gap vortices feature oscillating tails that penetrate into the lattice bulk.

To ensure a fair comparison between the ring structure and the disclination lattice, we set the ring-like lattice to have the same parameters as the disclination lattice (i.e., $p = 8$ and $w = 0.5$). Our linear stability analysis reveals that vortices with $m = 1$ (or $m = 2$) in $\mathcal{C}_4$ (or $\mathcal{C}_5$) symmetric ring-like lattices are stable throughout their entire existence domain under the condition of focusing nonlinearity [see Figs. S9(a) and S9(c)]. Moreover, $\mathcal{C}_7$ symmetric ring-like lattices support stable vortices with $m = 1$ and $m = 2$ [see Figs. S9(e) and S9(f)], while $\mathcal{C}_8$ symmetric ring-like lattices support stable vortices with $m = 3$ [see Fig. S9(i)]. In the focusing medium, stable vortex solitons with $m = 1$ have not been found in $\mathcal{C}_N$ ($N > 4$) symmetric ring-like lattices [see Figs. S9(b), S9(d), and S9(g)]. Similar conclusions

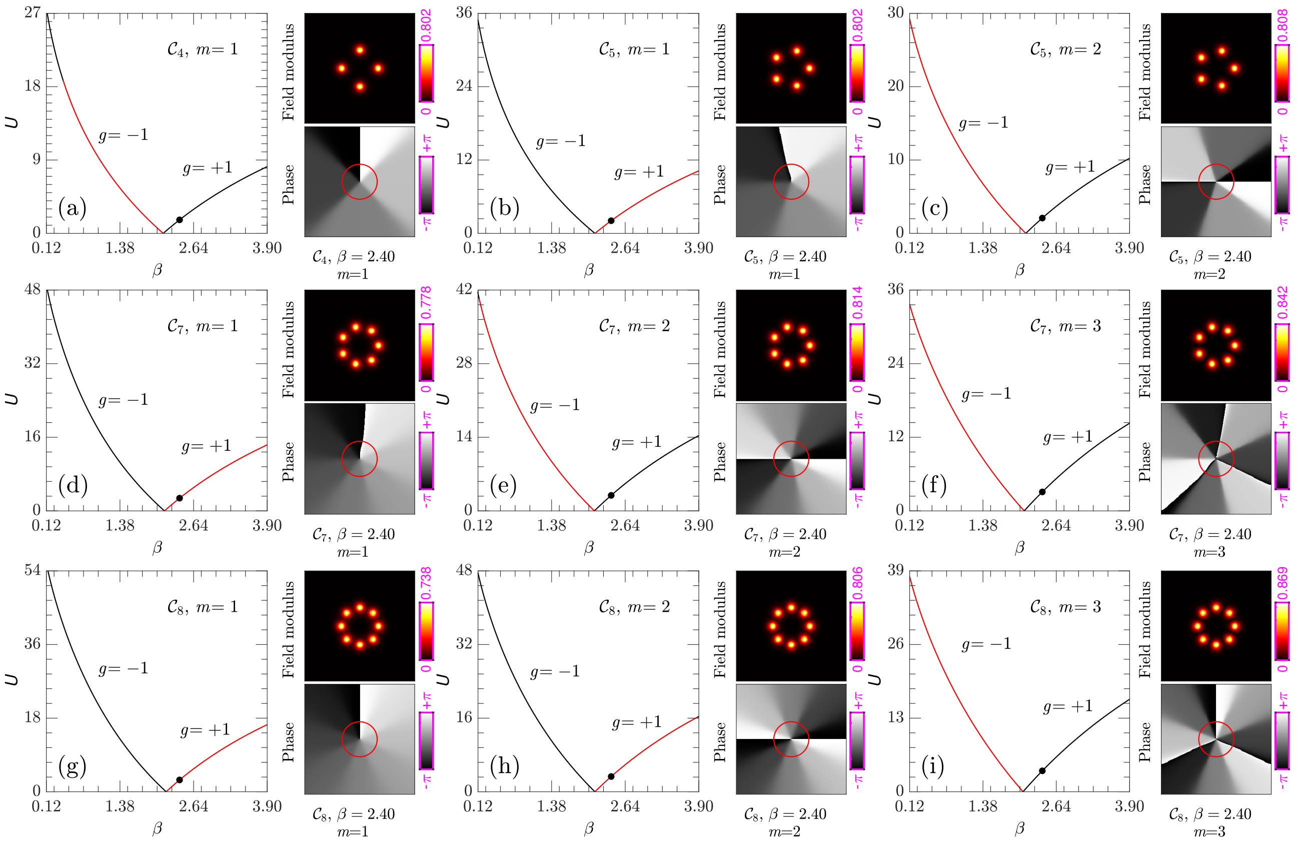

FIG. S8. Vortex solitons supported by ring-like lattices. The $U(\beta)$ curves for vortex solitons with $m = 1$, $\mathcal{C}_4$ lattice (a), $m = 1$, $\mathcal{C}_5$ lattice (b), $m = 2$, $\mathcal{C}_5$ lattice (c), $m = 1$, $\mathcal{C}_7$ lattice (d), $m = 2$, $\mathcal{C}_7$ lattice (e), $m = 3$, $\mathcal{C}_7$ lattice (f), $m = 1$, $\mathcal{C}_8$ lattice (g), $m = 2$, $\mathcal{C}_8$ lattice (h), $m = 3$, $\mathcal{C}_8$ lattice (i), are plotted. Stable families are indicated by black lines, while unstable families are indicated by red lines. Examples of vortex solitons are attached to the side of their respective $U(\beta)$ panels. Phase singularity in the center of each pattern is highlighted by the red circle.

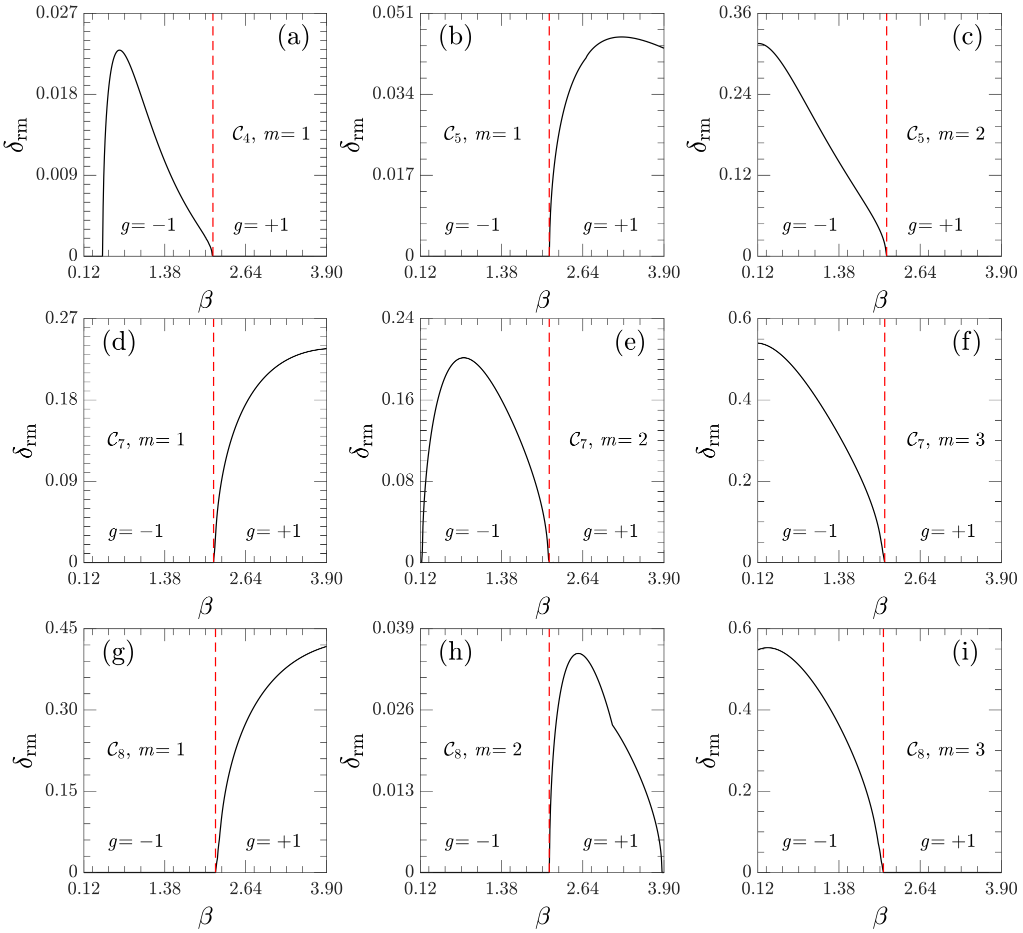

FIG. S9. The dependence of real part of the perturbation growth rate δ_{re} on the propagation constant β for vortex solitons supported by ring-like lattices. The $\delta_{\mathrm{re}}(\beta)$ curves for vortex solitons with $m = 1$, $\mathcal{C}_4$ lattice (a), $m = 1$, $\mathcal{C}_5$ lattice (b), $m = 2$, $\mathcal{C}_5$ lattice (c), $m = 1$, $\mathcal{C}_7$ lattice (d), $m = 2$, $\mathcal{C}_7$ lattice (e), $m = 3$, $\mathcal{C}_7$ lattice (f), $m = 1$, $\mathcal{C}_8$ lattice (g), $m = 2$, $\mathcal{C}_8$ lattice (h), $m = 3$, $\mathcal{C}_8$ lattice (i), are plotted. The red dashed line represents the boundary between focusing and defocusing nonlinearities.

have also been presented in Refs. [1, 2]. In the ring-like lattices, the flip of vortex stability under a change of sign of nonlinearity has been observed [3]. Comparing the stability of the vortex solitons in such ring-like structures with their counterpart in disclination latices, one immediately finds the stability between these two are entirely different, in other words, the stability of the vortex solitons in disclination lattices have been fundamentally altered by the presence of the waveguide arrays surrounding the core of the disclination lattices.

5, TOPOLOGICAL INDEX

The crystalline topology can be deduced from the symmetry indicators (band representations) [4]. For a higher-order topological insulator with a hexagonal unit cell, the primary topological index is [5]

$$\chi^{(6)} = \left(\left[M_1^{(2)}\right], \left[K_1^{(3)}\right]\right). \tag{S1}$$

where $\left[\Pi_p^{(n)}\right] \equiv \#\Pi_p^{(n)} - \#\Gamma_p^{(n)}$. Here $\#\Pi_p^{(n)}$ is the number of occupied bands at the high-symmetry point Π $(= \mathrm{M}, \mathrm{K})$ with the C_n rotation eigenvalues $e^{2\pi \mathrm{i}(p-1)/n}$ $(p = 1, ..., n)$. A disclination is characterized by the net translation (denoted by the Burgers vector B) and net rotation (denoted by the Frank angle Ω) accumulated under parallel transport of a vector along a loop enclosing the core. For the topologically nontrivial case ($\gamma = d_1/d_2 > 1$), one can find $\chi^{(6)} = (2, 0)$. While for the topologically trivial case ($\gamma = d_1/d_2 < 1$), $\chi^{(6)} = (0, 0)$. The secondary topological index of the disclination is given by

$$Q = \frac{\Omega}{2\pi}\left(\frac{3}{2}\left[M_1^{(2)}\right] - \left[K_1^{(3)}\right]\right) \bmod 1, \tag{S2}$$

yielding $Q = 0$ for a trivial phase and $Q = 3\Omega/2\pi$ mod 1 for a nontrivial phase.

---

[1] Y. V. Kartashov, A. Ferrando, A. A. Egorov, and L. Torner, Soliton topology versus discrete symmetry in optical lattices, Phys. Rev. Lett. **95**, 123902 (2005).
[2] A. S. Desyatnikov, M. R. Dennis, and A. Ferrando, All-optical discrete vortex switch, Phys. Rev. A **83**, 063822 (2011).
[3] P. Kevrekidis, H. Susanto, and Z. Chen, High-order-mode soliton structures in two-dimensional lattices with defocusing nonlinearity, Phys. Rev. E **74**, 066606 (2006).
[4] B. Bradlyn, L. Elcoro, J. Cano, M. G. Vergniory, Z. Wang, C. Felser, M. I. Aroyo, and B. A. Bernevig, Topological quantum chemistry, Nature **547**, 298 (2017).
[5] W. A. Benalcazar, T. Li, and T. L. Hughes, Quantization of fractional corner charge in C_n-symmetric higher-order topological crystalline insulators, Phys. Rev. B **99**, 245151 (2019).
